# Medication omission rates in New Zealand residential aged care homes: a national description

**DOI:** 10.1186/s12877-020-01674-w

**Published:** 2020-08-05

**Authors:** Stephanie. M. Garratt, Ngaire. M. Kerse, Kathryn Peri, Monique. F. Jonas

**Affiliations:** 1 School of Population Health, University of Auckland, Auckland, New Zealand; 2 National Ageing Research Institute, Melbourne, Australia; 3 School of Nursing, University of Auckland, Auckland, New Zealand

**Keywords:** Medication omissions, Nursing, Medication administration, Aged care, E-records

## Abstract

**Background:**

A medication omission is an event where a prescribed medication is not taken before the next scheduled dose. Medication omissions are typically classed as errors within Residential Aged Care (RAC) homes, as they have the potential to lead to harm if poorly managed, but may also stem from good clinical decision-making. This study aimed to quantify the incidence, prevalence, and types of medication omissions in RAC homes on a national scale, using a New Zealand-based sample.

**Methods:**

We conducted retrospective pharmacoepidemiology of de-identified medication administration e-records from December 1st 2016 to December 31st 2017. Four tiers of de-identified data were collected: RAC home level data (ownership, levels of care), care staff level data (competency level/role), resident data (gender, age, level of care), and medication related data (omissions, categories of omissions, recorded reasons for omission). Data were analysed using SPSS version 24 and Microsoft Excel.

**Results:**

A total of 11, 015 residents from 374 RAC homes had active medication charts; 8020 resided in care over the entire sample timeframe. A mean rate of 3.59 medication doses were omitted per 100 (±7.43) dispensed doses/resident. Seventy-three percent of residents had at least one dose omission. The most common omission category used was ‘not-administered’ (49.9%), followed by ‘refused’ (34.6%). The relationship between ownership type and mean rate of omission was significant (*p =* 0.002), corporate operated RAC homes had a slightly higher mean (3.73 versus 3.33), with greater variation. The most commonly omitted medications were Analgesics and Laxatives. Forty-eight percent of all dose omissions were recorded without a comment justifying the omission.

**Conclusions:**

This unique study is the first to report rate of medication omissions per RAC resident over a one-year timeframe. Although the proportion of medications omitted reported in this study is less than previously reported by hospital-based studies, there is a significant relationship between a resident’s level of care, RAC home ownership types, and the rate of omission.

## Background

Medication management and administration are essential services provided in residential aged care (RAC) homes, also called care homes or nursing homes. The more medications a resident is prescribed, the higher their risk of adverse events and poor outcomes [1]. Deviations from medication administration procedures can lead to medication errors, such as administering the wrong medication to the wrong resident [2].

Guidelines for medication administration in RAC homes, from prescribing to post-administration monitoring, commonly known as the ‘5 Rights plus 3,’ are set by professional organisations and prompt care staff to ensure the right medication and right dose, via the right route are given to the right person, at the right time [3, 4]. These rights and checks form the core of the medication administration process [3]. Three additional rights prompt staff to provide documentation, check the medication indication, and to consider a resident’s right to refuse medication [3]. Deviations from the guidelines occur, and may increase the risk of errors and adverse drug events [5]. Recording may not always reflect the reasons, clinical or otherwise, leading to an omission [6, 7].

A medication omission is often defined as a dose of medication that has been prescribed but not administered by the time of the next scheduled dose [1]. Previous studies on medication errors and omissions in acute hospitals, palliative care and residential aged care homes reported logistical limitations in sample size due to the resources needed for retrospective paper-based medication chart reviews across multiple sites [2, 5, 6, 8–10]. Furthermore, these studies have used a variety of categories of omissions, and have therefore reported omissions in different ways, influencing the way rates of omission are reported [1, 8].

To our knowledge there have been no population-level studies of medication omissions in RAC homes. This study aimed to quantify the incidence, prevalence, and categories of medication omission in RAC homes on a national-level, using a New Zealand database of electronic medication records (e-records). E-records are employed widely across New Zealand through medication systems such as Medi-Map™, which enable large-scale, de-identified, retrospective medication chart reviews [7, 11]. At time of publication, approximately 70% of all RAC homes in New Zealand operated the Medi-Map™ system.

## Methods

A retrospective review of de-identified medication e-records for residents in RAC homes across New Zealand from December 1st, 2016 to December 31st, 2017 was performed. This secondary data was sourced from Medi-Map Ltd., which provides software as a service to RAC homes in New Zealand and Australia. RAC homes were eligible for inclusion if they had used Medi-Map Ltd. for the entire year, with at least one-month of use prior to the study commencing. Ethics approval was provided by the University of Auckland Human Participants Ethics Committee (UAHPEC), Reference 020110. There were four levels of data collected from the Medi-Map™ database, presented in Table 1.

**Table 1:** Data collected from Medi-Map™ sample

RAC home level data	RAC staff data	RAC resident data	Medications data
Numerical ID RAC home ownership type	Numerical Staff ID Staff role (e.g. Registered Nurse)	Numerical Resident ID Level of care Age at time of data extraction Gender	Regular medications dispensed, administered, omitted Short course medications dispensed, administered, omitted. Reason for medication omission Omission category recorded (Refused, Withheld, Not-administered)

Residents were included if they had had medication administered by care staff during the time frame—either as a short course medication, or a regular medication administered for all or part of the timeframe. Regular and short course medications were included; Pro Re Nata (PRN) medications (medications charted to be given as needed/requested) were excluded. All categories of omission were included, based on the Medi-Map™ recording system: Refused (resident-initiated decision), Withheld (clinical/staff-initiated decision), and Not-Administered (medication or resident not available, or to be given later).

Level of care was coded into seven categories using care type funding levels (Table 1). Hospital Care is high-dependency complex care where residents need 24-hour nursing care; Rest Home Care is low-dependency care where residents need care daily but are semi-independent. Transitional Care includes shorter-stay residents, as well as those admitted to a RAC home for respite purposes. Palliative Care is a care funding classification for residents with a life limiting illness of less than 6 months, where quality of life and relief of suffering are the key concerns. There were two care funding levels for dementia—Dementia Residential, residents that require a locked door and 24-hour supervision but are semi-independent; and Dementia Hospital, for those with dementia who also require 24-hour nursing care. Young Person with Disability (YPD) refers to individuals under the age of 65 years who require long-term assistance and/or nursing care.

Each staff member that uses Medi-Map™ is assigned a competency level based on their qualifications. Staff competency levels for medication administration include: Manager, Registered Nurse, Enrolled Nurse, Bureau Nurse, Internationally Qualified Nurse, and Healthcare Assistant.

SPSS version 24 and Microsoft Excel were used to analyse demographic details including residents’ age and gender, care staff competency levels, medications omitted, and rates of omission. A proportion of residents had had their ages incorrectly recorded by their RAC home, leading to 453 residents with recorded ages from − 17 to 40. These 453 residents with an incorrect age were excluded from age analyses.

Descriptive statistics were used to describe the sample, the proportion of residents who had any omissions, and the types of medications omitted. The denominator for analyses was the resident. The incidence of omissions was calculated by the number of omissions / the total number of doses dispensed. The rate of medication omissions was generated per 100 dispensed doses of medication overall. Relationships between omissions, resident gender, level of care, and ownership status of RAC homes were tested using chi-squares, one-way ANOVA, independent samples T-tests, and a Kruskal-Wallis H test/Ranks Mann-Whitney.

## Results

The sample included approximately 60% (*n* = 374) of all registered RAC homes operating in New Zealand during 2017. In total, the sample contained 11,015 RAC home residents, with a sub-sample of 8020 residents that resided in RAC homes for the entire timeframe.

**Fig. 1:**
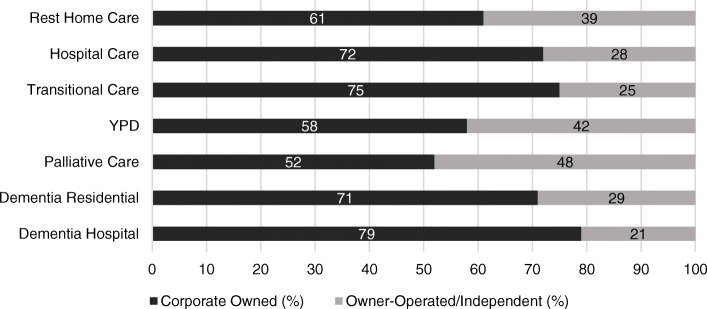
Proportion of Residents within Each Care Level, by RAC Home Ownership

The median age of residents was 87 years (range = 46–109 years). Sixty-eight percent (*n* = 7490) were female. Of the residents who had resided within care homes for the entire timeframe, 48% (*n* = 5286) were classified as rest home level care (Table 2). Sixty-six percent of all RAC homes were owned as part of a corporate entity, or large organization. The details with regard to RAC home ownership and each care level are presented in Fig. 1.

**Table 2:** Proportion of residents within each care level

Level of Care	Proportion of Residents
Hospital Care	3250 (29.5%)
Rest Home Care	5286 (48%)
Transitional Care	1255 (11.4%)
YPD	26 (0.2%)
Palliative Care	233 (2.1%)
Dementia Residential	776 (7.0%)
Dementia Hospital	143 (1.3%)
No Care Level specified	46 (0.4%)

### Medication omissions

All residents had had at least one regular medication administered over the Dec 2016 to Dec 2017 timeframe. Twenty-seven percent (*n* = 2975) of these residents had zero medication omissions (all medications administered).

Of the 31,921,548 individual medications doses dispensed to RAC home residents, a total of 934,753 doses were omitted (2.93% of all doses). The average number of dispensed medication doses was 9.87 medication doses per resident per day. The most commonly selected category of omission, ‘not administered’, accounted for 50% of all omissions. ‘Refused’, accounted for 34.6% of all recorded omissions and ‘Withheld’ medications accounted for 15.5%. Seventy-two percent of residents within the 11,015 individual sample had at least one dose omission during the period. All residents in care for the entire timeframe (*n* = 8020) had at least one dose omission.

Registered nurses were the primary recorders of medication omissions (60.7%), across all omission subcategories. Health care assistants certified to administer medications recorded 26.7% of all omissions overall, and 63.8% of all omissions classified as not-administered.

There was no association found between age, gender, and medication omission rate. Spearman’s correlation coefficient r_s_ for age and omissions was − 0.017 and not statistically significant (*p* = 0.130), with 33.8% males compared to 35% of females refusing at least one dose (n.s).

### Rates of omission

We used the group of 8020 residents who resided in a RAC home for the entire time to calculate rates of total omissions per 100 dispensed doses per resident, as well as for each of the three categories of omissions (Table. 3). The mean omission rate was 3.59 medications per 100 dispensed doses per person (s.d. 7.43). The high standard deviations for all rates indicate high variation between RAC home residents based on care level.

**Table 3:** Mean omission rate per 100 dispensed doses per resident & omission rate per omission category

Care Level	Omissions Rate per 100 doses Mean (s.d.)	Withheld Rate per 100 doses Mean (s.d.)	Refused Rate per 100 doses Mean (s.d.)	Not Administered Rate per 100 doses Mean (s.d)
Hospital Care	3.37 (5.71)	0.59 (1.29)	1.47 (4.44)	1.31 (2.50)
Rest Home Care	3.25 (7.22)	0.39 (1.48)	1.03 (4.09)	1.83 (5.17)
Transitional Care	8.49 (15.52)	0.49 (1.28)	1.80 (6.84)	6.20 (13.77)
YPD	2.47 (3.34)	0.29 (0.58)	0.98 (2.70)	1.20 (1.37)
Palliative Care	10.47 (11.63)	2.34 (6.57)	2.32 (3.97)	5.82 (8.90)
Dementia Residential	3.54 (7.82)	0.56 (1.79)	1.37 (4.91)	1.61 (4.72)
Dementia Hospital	3.66 (6.18)	1.19 (1.64)	1.36 (3.43)	1.11 (4.19)
Total	3.59 (7.43)	0.51 (1.55)	1.27 (4.43)	1.82 (5.17)

Level of care had a significant association with medication omissions rates (*p* = 0.00). Palliative care residents consistently had a higher omission rate than rest home residents (10.5 doses/100 doses/resident (s.d. 11.6), compared with 3.3 doses/100 doses/resident (s.d. 7.2) for Rest Home). The two dementia care levels had similar not-administered and refused rates per 100 dispensed doses/resident. However, residents in Dementia Residential had a lower withheld rate, and a slightly lower overall omission rate. Hospital level care residents had a higher rate of refused medication (1.47 (s.d. 4.44) when compared to Rest Home care residents (1.03 (s.d. 4.09). The highest omission rates were obtained for the category “not-administered” in five out of seven care levels (Table 3).

### RAC home ownership and omission rates

Sixty-six percent of the RAC home sample were corporate-owned (i.e. governed by a multi-facility organization). Corporate status was significantly associated with medication omission rates. Using an independent samples t-test (*p* = 0.025) and a Kruskal-Wallis H test, X^2^(2) = 10.065, *p* = 0.002, corporate RAC homes had a slightly higher rate of omissions (3.73 /100 doses/resident (s.d. 7.79) (*n* = 4064 omissions) versus 3.33 /100 doses/ resident (s.d. 6.65) (*n* = 3890 omissions) for independent facilities). Corporate RAC homes accounted for 71% of Dementia Residential and 79% of Dementia Hospital care, as well as 72% of all Hospital care (Fig. 1.). The rate of omissions classified as not-administered, per 100 dispensed doses/resident was higher for residents in corporate RAC homes, with a rate of 1.96 (s.d. 6.65) versus 1.54 (s.d. 4.05). Rates of refused and withheld medications were similar between the two ownership types (Table 4).

**Table 4:** Omissions rate per 100 doses per resident, by RAC home ownership

Ownership Type	Mean Omissions Rate per 100 doses (s.d.)	Mean Withheld Rate per 100 doses (s.d.)	Mean Refused Rate per 100 doses (s.d.)	Mean Not Administered Rate per 100 doses (s.d)
Corporate Governed	3.73 (7.79)	0.49 (1.51)	1.28 (4.33)	1.96 (6.65)
Owner-Operated/ Independent	3.33 (6.65)	0.55 (1.62)	1.24 (4.61)	1.54 (4.05)

### Medications omitted

During the review period, Paracetamol 500 mg tablets (*n* = 5,799,047 doses) were the most dispensed medication, with 3.01% (*n* = 174,661) of these doses omitted. The top six omitted medications included three forms of laxatives (*n* = 3,401,670 doses): Docusate Sodium 50 mg + Sennoside B 8 mg (Laxol®^)^, Lactulose 3.34 g/5 mg liquid, and Lax-sachets. Laxol® was the second most commonly dispensed (*n* = 2,276,636 doses) and omitted medication (*n* = 120,815 doses (5.31%)), but the proportion of omitted doses of lactulose and lax-sachets was higher—9.08% of lactulose and 11.06% of lax-sachet doses were omitted.

Short course medication omissions were primarily doses of antibiotics. Flucloxacillin 500 mg capsules were the most frequently dispensed and administered short-course medication, with 5.04% of all Flucloxacillin 500 mg doses omitted (*n* = 84,066 doses administered). The antibiotic with the highest omitted proportion over the timeframe was Trimethoprim 300 mg, with 25,759 doses dispensed and 11.04% of these doses omitted. Forty-eight percent of all medication omissions were recorded without a clinical note/rationale for the omission. This is explored in subsequent papers; it is unclear if these dose omissions are the result of clinical decision-making, medication unavailability, or whether these medication doses have been delayed, or simply not given [7].

## Discussion

Within hospital studies and adverse incident reporting, medication omissions are portrayed as a significant systemic patient care and service delivery issue [8]. This study suggests that medication omissions are common within RAC homes, with 73% of residents having at least one dose omission during the period. However, the rate of omissions per 100 doses is low, and varies between levels of care.

This is the first study that reports on a large sample and provides robust and specific detail on medication omission rates. Hospital-based studies have found an omission rate per medication administration episode of up to 11%, also stating that one in nine doses were omitted [10]. This study has found that only 2.93% of dispensed medication doses were omitted over the timeframe, significantly fewer than indicated by previous studies. This is a significant finding, as RAC homes are the major site for medication administration to patients/residents outside of hospital ward settings. Hospital-based studies typically contain a large proportion of new admissions and refer to medication not being available as a key contributor to medication omissions. We would expect the medication omission rate to be less in RAC homes as they do not have the high volume of admissions, and individuals typically stay in RAC longer than in a hospital setting. This study sets out a clear mean omission rate per resident, 3.59 doses/100 doses (s.d. 7.43), a level of detail which previous studies have been unable to provide.

### Medication omissions & RAC ownership

The rapid ageing of the New Zealand population has led to an increase in large organizations and corporate entities governing and building RAC homes. Corporate-owned RAC homes make up 66% of the sample for this study, and prior studies have not considered ownership types in relation to medication omissions. Corporate-owned RAC homes had a slightly higher rate of omission, both in general, and for medication omissions classified as not-administered (Table 3). This may reflect a lack of a clear definition for the not-administered category within these RAC homes.

Reviews suggest that corporate entities and for-profit RAC homes may provide lower quality care than independent or non-profit sites [12]. While research mainly focussed on the United States sector [13], studies in Europe also show increased signs of neglect in for-profit facilities [14]. In New Zealand each independent RAC home is responsible for its own policies around medication administration and management, whereas corporate entities operate under a standard policy written by the wider organization [12]. It is unclear whether differences in omission rates between ownership types reflect variances in quality of care, or policies, and as such, we cannot justify omissions as a quality indicator, however our findings are consistent with international research suggesting variation in outcome related to ownership type of RAC homes.

### Categories of Omission & Recording Practices

Refusals and medication unavailability have commonly been identified as the top two categories/reasons for omission [9, 10]. Refusals indicate a resident’s decision not to take an offered dose. The reasons behind this can relate to residents exercising autonomy, their preferences; on the other hand, there may be no clear rationale, and the resident may refuse medication for reasons that care staff cannot ascertain. The best practice guidelines prompt care staff to attempt administration up to three times, and to record a reason for the refusal [3]. The relationship between omissions and errors requires more consideration, as a resident refusal does not imply a clinical error, despite resulting in a dose omission. Almost 35% of omissions were refusals, significantly higher than prior studies, with a mean rate of 1.27 doses per 100 dispensed doses/resident [5]. Higher refusal rates were found in palliative, transitional, and dementia care types. Refusals of medication may be accepted more in these settings—care staff may persist with administration more in residential and hospital settings.

Medications ‘withheld’ based on care staff decision-making were the least frequent category of omission recorded, with 15.5% of all omissions. The withheld medication rate per 100 dispensed doses was consistently between 0.29 and 0.59 (s.d. 1.28–1.64) doses, with higher rates in Dementia Hospital and Palliative Care (Table 2). However, widespread inconsistent recording of why medications were omitted hinders our ability to explain why these medications were withheld. Care staff have an obligation to record clinical decisions and reasons for omission, so that prescribers, other staff, and the residents themselves can access these reasons. Yet New Zealand’s best practice guidelines provide less guidance on how to make decisions and withhold medications, compared to resident refusals [3, 4]. This implies that omissions based on clinical decisions are not subject to the same level of scrutiny that resident-initiated omissions are. There is, however a fine balance between processes and improving safety culture [15]. Our study adds to the international literature on medication errors and omissions [16, 17].

The most prevalent category of omission was ‘not administered’, a category originally designed to indicate medication or resident unavailability. Prior studies have used up to 12 different categories of omission, many of which can be grouped under the three categories present in Medi-Map™. For example, ‘vomiting’ has been used as its own omission category, but in Medi-Map™ this falls under ‘withheld’, as this is typically a reason for staff to initiate an omission. However, the use of fewer categories requires staff to better record why an omission has occurred, as ‘not-administered’ is not enough clinically to justify the omission; the care team also need to know why it was not-administered. The use of a less bounded category by the data provider for this study allows us to highlight the need for clear definitions and the provision of a list of common reasons for not-administering a medication. Of all omissions, 50% were recorded as ‘not administered’, with the highest omission rates per 100 doses, particularly related to Rest Home Care, Dementia Residential, Palliative, YPD and Transitional Care.

There is no real way of knowing why these medications were not given. Supply issues have been highlighted as complex to fix [18]. The broadness and frequent use of the ‘not administered’ category indicates that staff may not be following up in situations where medication was not given. There are a lack of comments explaining why a medication has been ‘not-administered’, and if a medication was omitted then staff returned to administer it later, then this medication dose would have shifted to ‘administered’ and not been present in our dataset. The ‘not-administered’ category has been adopted as a ‘catch-all’—comments that are left refer to medications being withheld or refused, despite these being their own separate categories of omission. Providing a menu of common reasons alongside an option to record a novel reason may be useful within e-records and would save time from a care provision perspective. Failure to record this information can obstruct the provision of care, leading to miscommunication, heightened risk to residents, delays in ordering or following up prescribed medications. This is a case where technology can support enhanced professional practice and increase the quality of clinical care, if it is used to its full potential.

### Medications omitted

This study confirms that laxatives and mild pain relief are the most commonly omitted medications during medication administration. This is in line with prior studies of medication omissions and errors in palliative, RAC home, and hospital settings [5, 9]. The omission of specific medications in RAC homes in New Zealand will be further discussed in a subsequent paper, as they are an additional concern from a quality perspective.

### Use of electronic medication records to track omissions

Electronic medication records, like their paper predecessors, are only as good as the user. They facilitate large scale analysis of long-term medication and administration data but require consistent recording and adequate detailing. There appears to be a relationship between ownership of RAC homes and how omissions are recorded from a rate of omission and omissions type perspective. There also appears to be a relationship between a resident’s care level and rate of omissions. This indicates that organisational policy and the type of care a resident is receiving may have an influence on care staff’s decision-making when a medication has the potential to be omitted.

A review of the medication administration guidelines for RAC homes is needed, both within New Zealand and internationally, considering the widespread adoption and potential of e-records. In New Zealand, records tell us that between the years of 2016–2017, 72.7% of residents within the RAC home sample for this study experienced a medication omission. Yet the best practice guidelines barely acknowledge omissions as a concern during medication administration—except for refusals. Omissions are listed as a form of error, situated beside medications given at the wrong dose time [3, 4, 11]. Internationally, research into care staff perceptions of medication omissions is needed, as well as a review of how omissions are treated in Best Practice Guidelines. Further work on supply chain [18], repeat omissions [17], and organisational culture related to medication use would expand this area [15].

### Limitations

There were three predominant limitations to this study. First, that the secondary data only tracked the care level funding of residents upon admission to their care home, rather than care level changes over time. The needs of RAC Home residents are likely to increase over time, and care levels and funding naturally change to meet these needs over time. Second, the data collection parameters did not include data on why medications had been prescribed, limiting our ability to discuss why certain medications were prescribed more frequently than others. Third, the data collected only related to dispensing and administration of medication—as dispensing data, RAC home, and resident data were used as denominators we cannot be certain that the proportion of medication omissions is not an underestimation. An analysis of prescribing data would help to clarify this, as this study focused upon events at a RAC home level.

## Conclusions

Medication omissions occur regularly in RAC homes, although the rate is significantly lower than previously reported by hospital-based studies. These omissions are significant: they reflect challenging situations that require good clinical decision-making from care staff. These decisions encompass a wide range of factors including availability of the resident or medication, clinical judgements about whether a medication should be withheld, and judgements about how to respond to resident refusals. This study identifies the significant influence level of care and RAC home ownership have on omission rates, and that omissions are not consistently recorded by care staff during medication rounds. Not all omissions may lead to adverse outcomes but if recorded poorly they may increase the chance of adverse outcomes and miscommunication.

## Data Availability

The datasets generated and analysed during the current study are not publicly available due to the nature of the raw data itself. This study uses private medication administration records that were de-identified by the data owner, Medi-Map Ltd., a private company operating in Australasia. Queries about the data source should be directed to office@medi-map.co.nz or https://medimap.co.nz/contact/

## Abbreviation

RAC Home: Residential Aged Care Home
